# 3′-Sialyllactose Protects SW1353 Chondrocytic Cells From Interleukin-1β-Induced Oxidative Stress and Inflammation

**DOI:** 10.3389/fphar.2021.609817

**Published:** 2021-04-12

**Authors:** Ahreum Baek, So Hee Jung, Soonil Pyo, Soo Yeon Kim, Seongmoon Jo, Lila Kim, Eun Young Lee, Sung Hoon Kim, Sung-Rae Cho

**Affiliations:** ^1^ Department of Rehabilitation Medicine, Yonsei University Wonju College of Medicine, Wonju, Korea; ^2^ Department and Research Institute of Rehabilitation Medicine, Yonsei University College of Medicine, Seoul, Korea; ^3^ Department of Rehabilitation Medicine, The Graduate School Yonsei University Wonju College of Medicine, Wonju, Korea; ^4^ Brain Korea 21 PLUS Project for Medical Science, Yonsei University College of Medicine, Seoul, Korea; ^5^ Department of Medicine, Yonsei University Wonju College of Medicine, Wonju, South Korea; ^6^ GeneChem Inc., Daejeon, Korea; ^7^ Graduate Program of Nano Science and Technology, Yonsei University College of Medicine, Seoul, Korea; ^8^ Rehabilitation Institute of Neuromuscular Disease, Yonsei University College of Medicine, Seoul, Korea

**Keywords:** osteoarthritis, 3′-sialyllactose, oxidative stress, inflammation, apoptosis, matrix metalloproteinases

## Abstract

Osteoarthritis (OA) is a major degenerative joint disease. Oxidative stress and inflammation play key roles in the pathogenesis of OA. 3′-Sialyllactose (3′-SL) is derived from human milk and is known to regulate a variety of biological functions related to immune homeostasis. This study aimed to investigate the therapeutic mechanisms of 3′-SL in interleukin-1β (IL-1β)-treated SW1353 chondrocytic cells. 3′-SL potently suppressed IL-1β-induced oxidative stress by increasing the levels of enzymatic antioxidants. 3′-SL significantly reversed the IL-1β mediated expression levels of reactive oxygen species in IL-1β-stimulated chondrocytic cells. In addition, 3′-SL could reverse the increased levels of inflammatory markers such as nitrite, prostaglandin E2, inducible nitric oxide synthase, cyclooxygenase-2, IL-1β, and IL-6 in IL-1β-stimulated chondrocytic cells. Moreover, 3′-SL significantly inhibited the apoptotic process, as indicated by the downregulation of the pro-apoptotic protein Bax, upregulation of the anti-apoptotic protein Bcl-2 expression, and significant reduction in the number of TUNEL-positive cells in the IL-1β-treated chondrocytic cells. Furthermore, 3′-SL reversed cartilage destruction by decreasing the release of matrix metalloproteinases (MMP), such as MMP1, MMP3, and MMP13. In contrast, 3′-SL significantly increased the expression levels of matrix synthesis proteins, such as collagen II and aggrecan, in IL-1β-treated chondrocytic cells. 3′-SL dramatically suppressed the activation of mitogen-activated protein kinases (MAPK) and phosphatidylinositol-3-kinase (PI3K)/protein kinase B (AKT)/nuclear factor kappa-light-chain-enhancer of activated B cells (NF-κB) signaling pathways, which are related to the pathogenesis of OA. Taken together, our data suggest that 3′-SL alleviates IL-1β-induced OA pathogenesis via inhibition of activated MAPK and PI3K/AKT/NF-κB signaling cascades with the downregulation of oxidative stress and inflammation. Therefore, 3′-SL has the potential to be used as a natural compound for OA therapy owing to its ability to activate the antioxidant defense system and suppress inflammatory responses.

## Introduction

Osteoarthritis (OA) is a complex progressive degenerative joint disorder that accompanies cartilage degradation and physical disability (Sanchez et al., 2005; Sanchez et al., 2008; Jeon et al., 2017). The development and progression of OA are related to oxidative stress-induced cartilage damage and an imbalance between catabolic and anabolic factors in joints (Loeser, 2009; Appleton, 2018). Although the occurrence and development of OA have been studied extensively, there is currently no efficient therapy to prevent OA progression.

The accumulation of reactive oxygen species (ROS) causes an increase in oxidative stress, and these reactive products are detoxified by the anti-oxidative defensive system (Betteridge, 2000; Jones, 2008). ROS are free radicals that are mainly generated by mitochondria, in the form of non-mitochondrial membrane-bound nicotinamide adenine dinucleotide phosphate (NADPH) oxidase and xanthine oxidase (XO) (Turrens, 2003). Several studies have indicated that elevated oxidative stress and excessive generation of ROS are observed in OA patients (Altindag et al., 2007; Erturk et al., 2012; Altay et al., 2015).

Recent studies revealed that excessive generation of ROS occurs as OA develops, leading to increased inflammation (Bolduc et al., 2019; Xie et al., 2019; Ansari et al., 2020). Interleukin-1β (IL-1β) and IL-6 are highly upregulated in OA joints and play important roles in the pathogenesis of OA by modulating oxidative stress, apoptosis, cartilage extracellular matrix degradation extracellular matrix (ECM) synthesis, and intracellular signaling pathways in OA (Kapoor et al., 2011; Lepetsos and Papavassiliou, 2016; Collins et al., 2018). In particular, mitogen-activated protein kinases (MAPK) and phosphoinositol 3 kinase (PI3K)/protein kinase B (AKT)/nuclear factor-κ light chain enhancer of activated B cells (NF-κB) pathways play vital roles in OA pathogenesis (Ahmed et al., 2005; Lu et al., 2018; Huang et al., 2019). Thus, oxidative stress and inflammation are closely related, and their regulation should be considered as therapeutic strategies of OA.

Human breast milk contains various bioactive factors with developmental and protective functions (Gila-Diaz et al., 2019). 3′-Sialyllactose (3′-SL) contains N-acetyl-D-neuramic acid and galactose subunit of lactose (Luo et al., 2014). 3′-SL is known to regulate a variety of biological functions in immune homeostasis (Zenhom et al., 2011; Donovan and Comstock, 2016). Moreover, 3′-SL has demonstrated therapeutic effects in OA and rheumatoid arthritis by protecting cartilage degradation and modulating chemokines and cytokines, respectively (Jeon et al., 2018; Kang et al., 2018). However, the antioxidant and inflammatory activities of 3′-SL in OA remain uncharacterized.

Human SW1353 chondrocytic cells and human chondrocytes have similar phenotypes. Previous studies have shown that IL-1β can mimic the pathological microenvironment of OA chondrocytes (Jia et al., 2013; Bao et al., 2016). Herein, we investigated the antioxidant and anti-inflammatory activities and mechanisms of action of 3′-SL on IL-1β-treated human SW1353 chondrocytic cells and explored the mechanisms underlying potential therapeutic effects in OA.

## Materials and Methods

### Reagents

SW1353 human chondrocytic cells were obtained from American Type Culture Collection (ATCC HTB-94; Manassas, VA, United States). Dulbecco’s modified Eagle medium with high glucose medium (DMEM-HG) was obtained from Hyclone (Grand Island, NY, United States). Fetal bovine serum (FBS) was purchased from T&I (Seoul, Korea). Phosphate-buffered saline (PBS) was provided by Welgene (Daegu, Korea). Penicillin-streptomycin, trypsin-EDTA, and BCA™ Protein Assay Kit were obtained from Thermo Fisher Scientific (Waltham, MA, United States). 3′-SL was provided by GeneChem Inc (Daejeon, Korea). Recombinant human IL-1β protein, nitrite, prostaglandin E2 (PGE2), IL-1β, and IL-6 ELISA kits were purchased from R&D Systems (Minneapolis, MN, United States). Nuclear factor kappa-light-chain-enhancer of activated B cells (NF-κB) inhibitor Bay 11-7082, dimethyl sulfoxide, and gelatin were obtained from Sigma-Aldrich (Saint Louis, MO, United States). Total antioxidant capacity (TAC) assay kit, 2′,7′-dichlorofluorescin diacetate (DCFDA)-cellular ROS assay kit, superoxide dismutase (SOD) activity assay kit, catalase activity assay kit, and oxidative stress defense cocktail were purchased from Abcam (Cambridge, MA, United States). DeadEnd™ Fluorometric Terminal dUTP Nick End-Labeling (TUNEL) System and 4,6-diamidino-2-phenylindole (DAPI; Vectorshield were purchased from Promega (Madison, WI, United States) and Vector Laboratories (Burlingame, CA, United States), respectively. TRIzol reagent, 4–12% Bis-Tris gels, 1× NuPage MES SDS running buffer, MOPS SDS running buffer, PVDF membrane, and NuPage Transfer Buffer were obtained from Invitrogen Life Technologies (Carlsbad, CA, United States). ReverTra Ace^®^ qPCR RT Master Mix with gDNA Remover and qPCRBIO SyGreen Mix Hi-ROX were purchased from Toyobo (Osaka, Japan) and PCR BIOSYSTEMS (London, United Kingdom), respectively. Bax, Bcl-2, iNOS, COX-2, MMP1, MMP3, MMP13, collagen II, aggrecan, p-extracellular signal-regulated kinase (ERK), ERK, p-P38, P38, p-c-Jun N-terminal kinase (JNK), JNK, p-PI3K, PI3K, p-AKT, AKT, p-inhibitor of nuclear factor kappa B (p-IκBα), IκBα, p-P65, P65, glyceraldehyde-3-phosphate dehydrogenase (GAPDH), and horseradish peroxidase–conjugated secondary antibodies were obtained from Santa Cruz Biotechnology (Dallas, TX, United States). p-PI3K antibody and enhanced chemiluminescence detection system were purchased from Cell Signaling Technologies (Beverly, MA, United States) and Amersham Pharmacia Biotech (Little Chalfont, United Kingdom), respectively.

### Cell Culture

Human chondrocytic cells were maintained in DMEM-HG with 10% FBS and 1% penicillin-streptomycin at 37°C. When approximately 80% confluency was achieved, the cells were washed with PBS and harvested with 0.05% trypsin-EDTA. Next, the cells were washed, centrifuged (1,000 g, 5 min, 25°C), resuspended, and finally seeded in new plates. The medium was replaced every 2–3 days. The cells were treated with IL-1β (10 ng/ml) under hypoxic conditions for 24 h, as previously described (Baek et al., 2018a; Baek et al., 2018b) to mimic the pathological microenvironment of OA chondrocytes. Then, 50 or 100 μM of 3′-SL was added and incubation continued for 24 h. In the NF-κB inhibitor experiment, the cells were pretreated with Bay 11-7082 (10 µM), which was dissolved in dimethyl sulfoxide, before the cells were exposed to IL-1β with or without 3′-SL. The culture supernatants were harvested, centrifuged (2,000 g, 5 min, 25°C), and stored at −70°C until further analysis. The cell pellets were washed with ice-cold PBS, centrifuged (2,000 g, 5 min, 4°C), and stored at −70°C until further analysis.

### Enzyme-Linked Immunosorbent Assay

Cell culture supernatants were collected, centrifuged (12,000 ×g, 5 min, 4°C), and stored at −70°C until use. The production of ROS, activities of TAC, SOD, and CAT, and the levels of IL-1β, IL-6, nitrite, and PGE2 in cell culture supernatants were measured according to the manufacturer’s instructions.

### Terminal dUTP Nick End-Labeling Assay

For analysis of apoptosis, SW1353 human chondrocytic cells were seeded in gelatin-coated slides in 6-well cell plates. IL-1β and 3′-SL treatments were performed as described in *Cell Culture*. The DeadEnd™ Fluorometric TUNEL System was conducted according to the manufacturer’s protocol. Samples were mounted on glass slides with fluorescent mounting medium with DAPI for imaging, using the Zeiss Axio Imager M2 (Carl Zeiss, Gottingen, Germany) fluorescence microscope. The number of positively stained cells over the total number of cells per specimen field was measured, and the percentage of positive cells was calculated. Four individual specimens per group were analyzed.

### Quantitative Real-Time Reverse Transcription Polymerase Chain Reaction

Total RNA extraction from the cell pellets was carried out using the TRIzol reagent, according to the manufacturer’s protocol. Total RNA was used in cDNA synthesis with ReverTra Ace^®^ qPCR RT Master Mix with gDNA Remover. The mRNA expression of genes such as iNOS, COX-2, MMP1, MMP3, MMP13, collagen II, aggrecan, and GAPDH was profiled with qPCRBIO SyGreen Mix Hi-ROX in a StepOnePlus Real-Time PCR System (Applied Biosystems, Foster City, CA, United States). Data analysis was performed using the 2^-ΔΔCT^ method. Primers used for qRT-PCR are described in Table 1.

**TABLE 1:** Primers used for qRT-PCR.

Gene symbol	Forward primer (5′→3′)	Reverse primer (5′→3′)
*iNOS*	AGG​GAC​AAG​CCT​ACC​CCT​C	CTC​ATC​TCC​CGT​CAG​TTG​GT
*COX2*	CTG​GCG​CTC​AGC​CAT​ACA​G	CGC​ACT​TAT​ACT​GGT​CAA​ATC​CC
*MMP1*	GGG​GCT​TTG​ATG​TAC​CCT​AGC	TGT​CAC​ACG​CTT​TTG​GGG​TTT
*MMP3*	CTG​GAC​TCC​GAC​ACT​CTG​GA	CAG​GAA​AGG​TTC​TGA​AGT​GAC​C
*MMP13*	TCC​TGA​TGT​GGG​TGA​ATA​CAA​TG	GCC​ATC​GTG​AAG​TCT​GGT​AAA​AT
*COL2A*	TGG​ACG​CCA​TGA​AGG​TTT​TCT	TGG​GAG​CCA​GAT​TGT​CAT​CTC
*Aggrecan*	GTG​CCT​ATC​AGG​ACA​AGG​TCT	GAT​GCC​TTT​CAC​CAC​GAC​TTC
*GAPDH*	AAGGGTCATCATCTCTGCCC	GTGAGTGCATGGACTGTGGT

### Western Blot Analysis

To assess the oxidative stress defense WB cocktail, Bax, Bcl-2, iNOS, COX-2, MMP1, MMP3, MMP13, collagen II, aggrecan, p-ERK, ERK, p-P38, P38, p-JNK, JNK, p-PI3K, PI3K, p-AKT, AKT, p-IκBα, IκBα, p-P65, P65, and GAPDH. The proteins from cell pellets were harvested and quantified using the BCA™ Protein Assay Kit. The protein samples were denatured and separated in 4–12% Bis-Tris gels with 1× NuPage MES and MOPS SDS running buffer. Proteins were transferred onto a PVDF membrane in NuPage Transfer Buffer with methanol at 4°C. The membranes were blocked with TBS plus Tween 20 in 5% skim milk and then incubated overnight at 4°C with the following primary antibodies: oxidative stress defense WB cocktail (1:250), Bax (1:1,000), Bcl-2 (1:1,000), iNOS (1:1,000), COX-2 (1:1,000), MMP1 (1:1,000), MMP3 (1:1,000), MMP13 (1:1,000), collagen II (1:1,000), Aggrecan (1:1,000), p-ERK (1:1,000), ERK (1:1,000), p-P38 (1:1,000), P38 (1:1,000), p-JNK (1:1,000), JNK (1:1,000), p-PI3K (1:1,000), PI3K (1:1,000), p-AKT (1:1,000), AKT (1:1,000), p-IκBα (1:1,000), IκBα (1:1,000), p-P65 (1:1,000), P65 (1:1,000), and GAPDH (1:1,000). The next day, the blots were washed three times with TBS plus Tween 20 and incubated with horseradish peroxidase–conjugated secondary antibodies (1:3,000) at 25°C for 1 h. After the blots were rinsed three times, the protein was detected with an enhanced chemiluminescence detection system (Amersham Pharmacia Biotech, Little Chalfont, United Kingdom). The relative expression of each protein was quantified using the internal controls (smooth muscle actin and GAPDH) or the total form of proteins (ERK, P38, JNK, PI3K, AKT, IκBα, and P65) with Multi Gauge (v3.0) software (Fujifilm, Tokyo, Japan).

### Statistical Analysis

All data are expressed as mean ± standard error of the mean from at least three independent experiments. Statistical analyses were conducted using the Statistical Package for Social Sciences (SPSS) version 25.0 (SPSS, Inc, Chicago, IL, United States). To confirm statistically significant results, one-way analysis of variance, followed by a post hoc Bonferroni comparison, was conducted. Statistical significance was set at *p* < 0.05.

## Results

### 3′-Sialyllactose Suppressed Interleukin-1β-Induced Oxidative Stress in the Chondrocytic Cells

**FIGURE 1:**
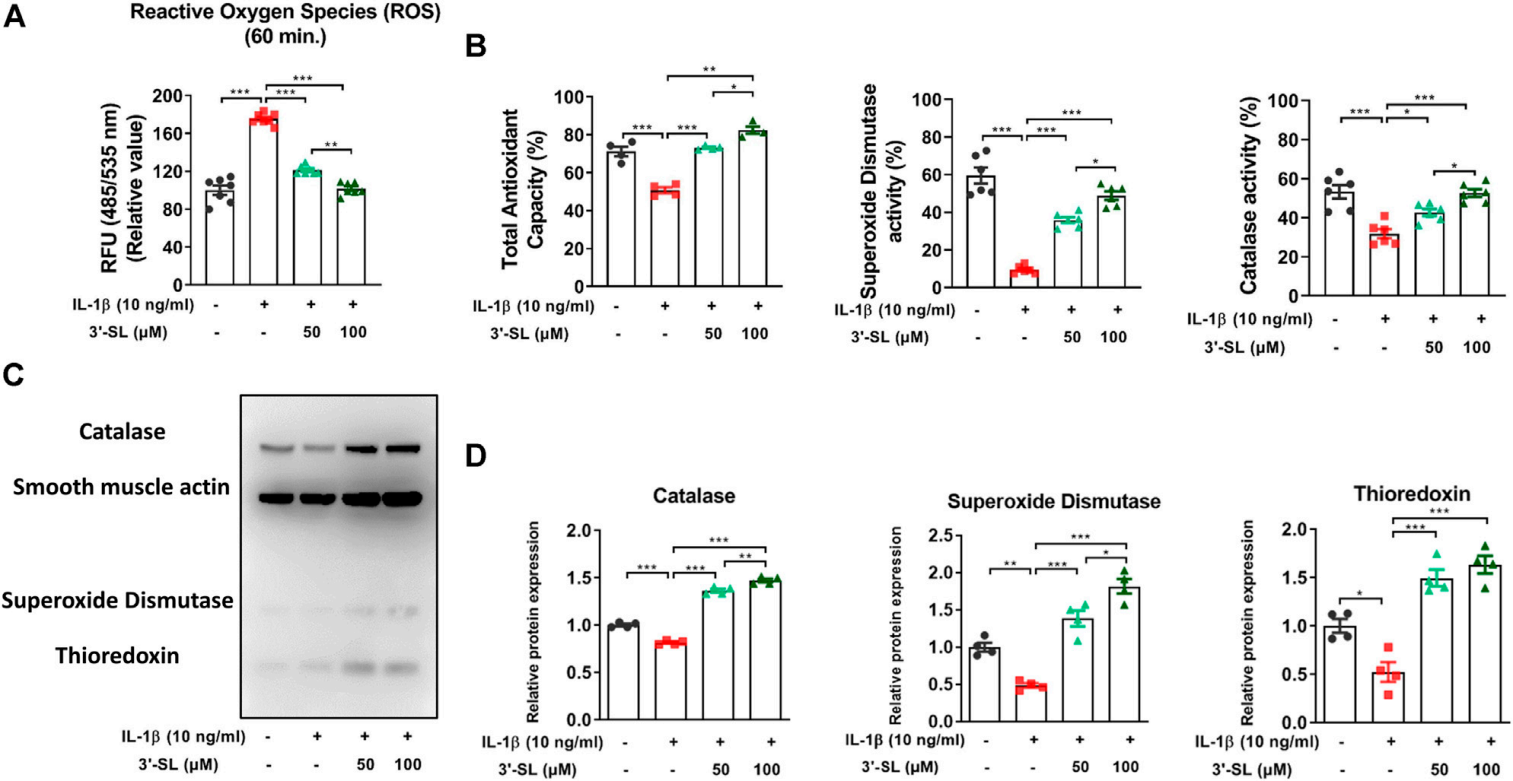
Effect of 3′-SL on IL-1β-induced oxidative stress in the chondrocytic cells. Chondrocytic cells were stimulated with or without IL-1β (10 ng/ml) for 24 h and then treated with the different concentrations of 3′-SL (50 and 100 μM) for 24 h. **(A)** Generation of ROS was measured using the DCFDA assay. **(B)** Antioxidant enzyme activities were analyzed using specific enzyme assay kits **(C)** The oxidative stress defense (Catalase, SOD1, TRX, smooth muscle actin) western blot cocktail was determined by western blot and **(D)** quantification analysis. The values are mean ± standard error of the mean. *p < 0.05, **p < 0.01, and ***p < 0.001. 3′-SL, 3′-Sialyllactose; IL-1β; interleukin-1β; ROS, reactive oxygen species; DCFDA, 2′,7′-dichlorofluorescin diacetate; SOD1, superoxide dismutase 1; TRX, thioredoxin.

To determine whether 3′-SL has antioxidant effects, generation of ROS in the chondrocytic cells was induced by IL-1β. Treatment with IL-1β elevated ROS levels in the chondrocytic cells. This elevation was significantly attenuated by treatment with 3′-SL in a dose-dependent manner (Figure 1A, **p < 0.01, and ***p < 0.001). Next, total antioxidant capacity and antioxidant enzyme activities were examined. 3′-SL potently suppressed IL-1β-induced oxidative stress, as revealed by the significant increase in total antioxidant capacity and levels of antioxidant enzymes, such as SOD and catalase, in a dose-dependent manner (Figures 1B–D, *p < 0.05, **p < 0.01, and ***p < 0.001). Similarly, thioredoxin, which was decreased by IL-1β, was significantly increased after 3′-SL treatment of the chondrocytic cells (Figures 1C,D, *p < 0.05, **p < 0.01, and ***p < 0.001). Taken together, these findings suggest that 3′-SL could suppress IL-1β-induced oxidative stress in chondrocytic cells via the reduction of ROS and the upregulation of antioxidant enzyme activities.

### 3′-Sialyllactose Suppressed Interleukin-1β-Induced Inflammatory Response in the Chondrocytic Cells

**FIGURE 2:**
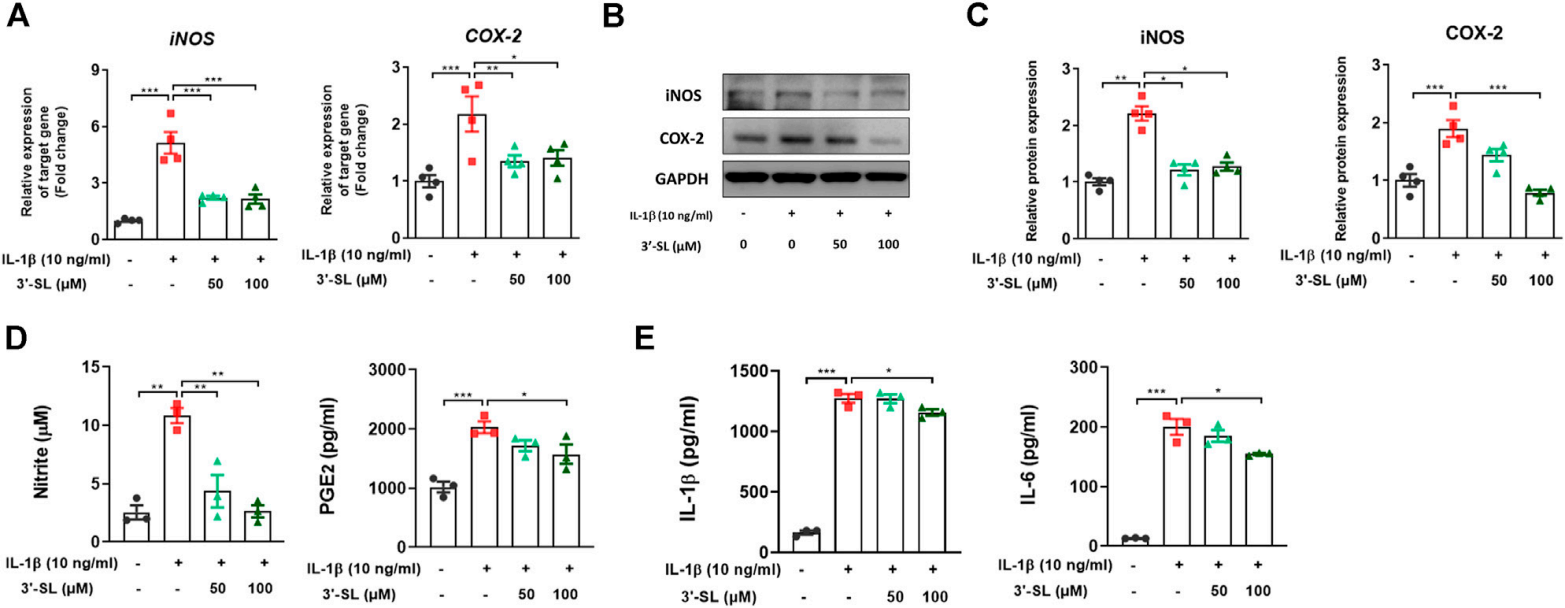
Effect of 3′-SL on IL-1β-induced inflammatory responses in the chondrocytic cells. Chondrocytic cells were stimulated with or without IL-1β (10 ng/ml) for 24 h and then treated with different concentrations of 3′-SL (50 and 100 μM) for 24 h. **(A)** The mRNA expression levels of iNOS and COX-2 were assayed by qRT-PCR. **(B)** The protein expression levels of iNOS and COX-2 were determined by western blot and **(C)** quantification analysis. **(D)** The nitrite levels in the culture medium were assessed by the Griess reaction. The levels of PGE2 were determined using ELISA. **(E)** The levels of IL-1β and IL-6 were determined using ELISA. The values are the mean ± standard error of the mean. *p < 0.05, **p < 0.01, and ***p < 0.001. 3′-SL, 3′-Sialyllactose; IL-1β; interleukin-1β; iNOS, inducible nitric oxide synthase; COX-2, cyclooxygenase-2; PGE2, prostaglandin E2.

To determine whether 3′-SL has the ability to act against the inflammatory response induced by IL-1β, the expression of inflammatory mediators was examined in chondrocytic cells. 3′-SL significantly reduced the increased mRNA and protein levels of iNOS and COX-2 (Figures 2A–C, *p < 0.05, **p < 0.01, and ***p < 0.001). Moreover, the production of endogenous nitrite and PGE2 was upregulated when cells were treated with IL-1β (Figure 2D, *p < 0.05, **p < 0.01, and ***p < 0.001). However, after treatment with 3′-SL, the production of nitrite and PGE2 was significantly downregulated (Figure 2D, *p < 0.05, **p < 0.01, and ***p < 0.001). Moreover, IL-1β treatment significantly increased IL-1β and IL-6 production (Figure 2E, *p < 0.05, and ***p < 0.001). The increased production of these molecules was reversed by treatment with 3′-SL. Taken together, these results indicate that 3′-SL could exert anti-inflammatory properties by inhibiting the inflammatory response in chondrocytic cells.

### 3′-Sialyllactose Suppressed Interleukin-1β-Induced Apoptosis in the Chondrocytic Cells

**FIGURE 3:**
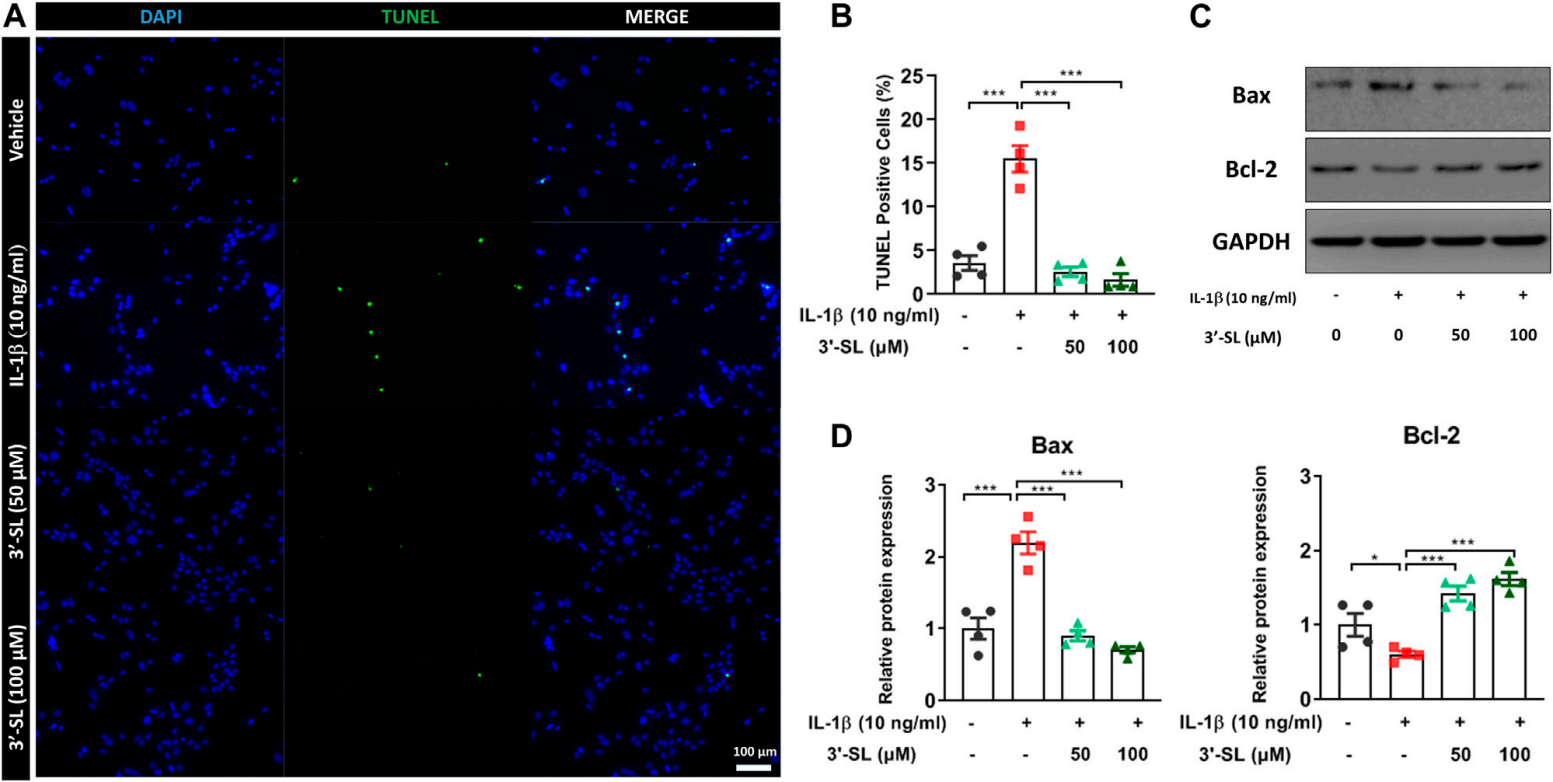
Effect of 3′-SL on IL-1β-induced apoptosis in the chondrocytic cells. Chondrocytic cells were stimulated with or without IL-1β (10 ng/ml) for 24 h and then treated with different concentrations of 3′-SL (50 and 100 μM) for 24 h. **(A)** Cell death was evaluated by Terminal deoxynucleotidyl transferase dUTP nick-end labeling (TUNEL) staining and representative images were shown. Scale bar: 100 μM. **(B)** The quantification of the number of TUNEL^+^ cells. **(C)** The protein expression levels of Bax and Bcl‐2 were determined by western blot and **(D)** quantification analysis. The values are the mean ± standard error of the mean. *p < 0.05, **p < 0.01, and ***p < 0.001. 3′-SL, 3′-Sialyllactose; IL-1β; interleukin-1β.

To investigate whether 3′-SL could inhibit IL-1β-induced apoptosis, terminal deoxynucleotidyl transferase dUTP nick-end labeling (TUNEL) staining and the expression of apoptosis-related proteins were examined in the chondrocytic cells. The percentage of TUNEL-positive cells was significantly upregulated in IL‐1β-treated chondrocytic cells (Figures 3A,B and ***p < 0.001). The percentage of TUNEL-positive cells was significantly decreased after treatment with 3'‐SL (Figures 3A,B and ***p < 0.001). Next, IL‐1β significantly increased the expression of the pro-apoptotic protein Bax and decreased the expression of anti-apoptotic Bcl-2 (Figures 3C,D, *p < 0.05, and ***p < 0.001). However, treatment with 3'‐SL significantly reversed these alterations (Figures 3C,D, **p < 0.01, and ***p < 0.001). Taken together, these results show that 3′-SL could reduce apoptosis in IL-1β-treated chondrocytic cells.

### 3′-Sialyllactose Suppressed Interleukin-1β-Induced Cartilage Matrix Degradation in the Chondrocytic Cells

**FIGURE 4:**
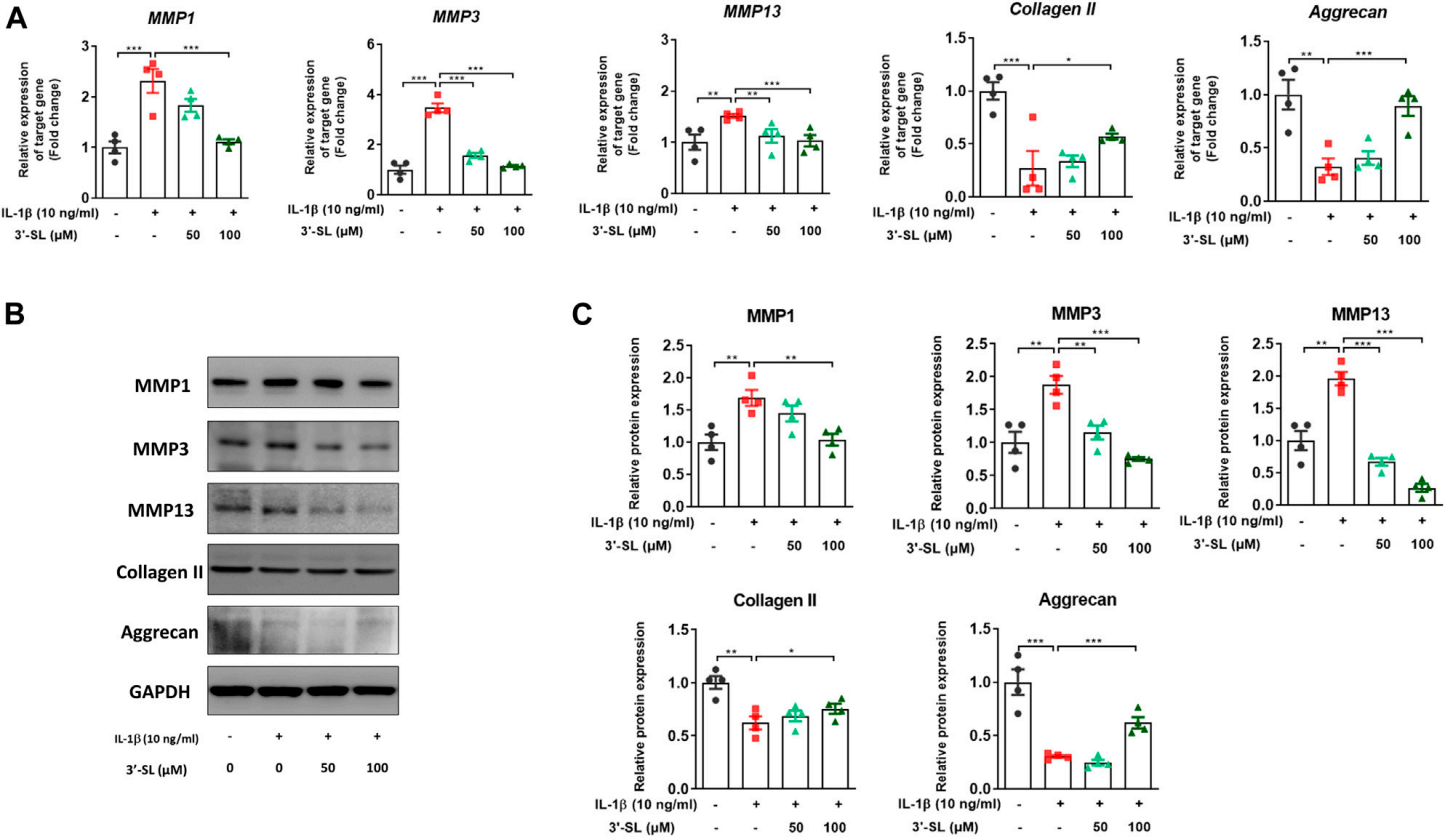
Effect of 3′-SL on IL-1β-induced cartilage destruction in the chondrocytic cells. Chondrocytic cells were stimulated with or without IL-1β (10 ng/ml) for 24 h and then treated with different concentrations of 3′-SL (50 and 100 μM) for 24 h. **(A)** The mRNA expression levels of MMP-1, MMP-3, MMP-13, collagen II, and aggrecan were assayed by qRT-PCR. **(B)** The protein expression levels of MMP-1, MMP-3, MMP-13, collagen II, and aggrecan were determined by western blot and **(C)** quantification analysis. The values are the mean ± standard error of the mean. *p < 0.05, **p < 0.01, and ***p < 0.001. 3′-SL, 3′-Sialyllactose; IL-1β; interleukin-1β; MMP, matrix metalloprotein.

To assess whether 3′-SL can prevent the production of MMPs, which are catabolic factors in OA pathogenesis, the expression of MMPs was examined in IL-1β-treated chondrocytic cells. 3′-SL significantly suppressed IL-1β-induced expression of MMP1, MMP3, and MMP13 (Figure 4, *p < 0.05, **p < 0.01, and ***p < 0.001). Collagen II and aggrecan are the two major components of the matrix, and they are considered as ECM synthesis genes. To explore whether 3′-SL mitigates IL-1β-induced ECM degradation in the cell model of OA, the expression of ECM synthesis genes was examined. As shown in Figure 4, IL-1β decreased the expression levels of collagen II and aggrecan, while 3′-SL treatment significantly suppressed IL-1β-induced cartilage degradation (*p < 0.05, **p < 0.01, and ***p < 0.001). Taken together, these results indicate that 3′-SL could inhibit cartilage matrix degradation by alleviating ECM degradation in IL-1β-induced chondrocytic cells.

### 3′-Sialyllactose Suppressed InterleukinL-1β-Induced Activation of Mitogen-Activated Protein Kinases and PI3K/AKT/NF-kB Signaling Pathways in the Chondrocytic Cells

**FIGURE 5:**
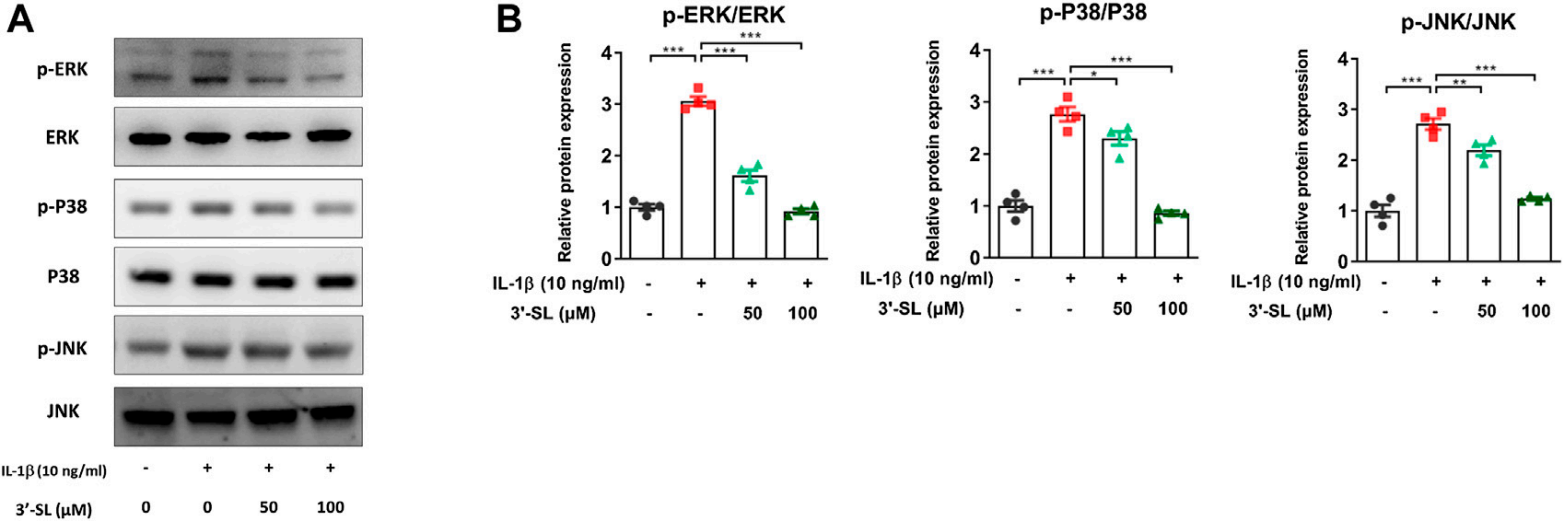
Effect of 3′-SL on IL-1β-induced MAPK signaling pathway in the chondrocytic cells. Chondrocytic cells were stimulated with or without IL-1β (10 ng/ml) for 24 h and then treated with the different concentrations of 3′-SL (50 and 100 μM) for 24 h. **(A)** The protein expression levels of p-ERK, ERK, p-P38, P38, p-JNK, and JNK were determined by western blot and **(B)** quantification analysis. *p < 0.05, **p < 0.01, and ***p < 0.001. 3′-SL, 3′-Sialyllactose; IL-1β; interleukin-1β; MAPK, Mitogen-activated protein kinase; ERK, extracellular signal-regulated kinase; JNK, c-JUN N-terminal kinase.

**FIGURE 6:**
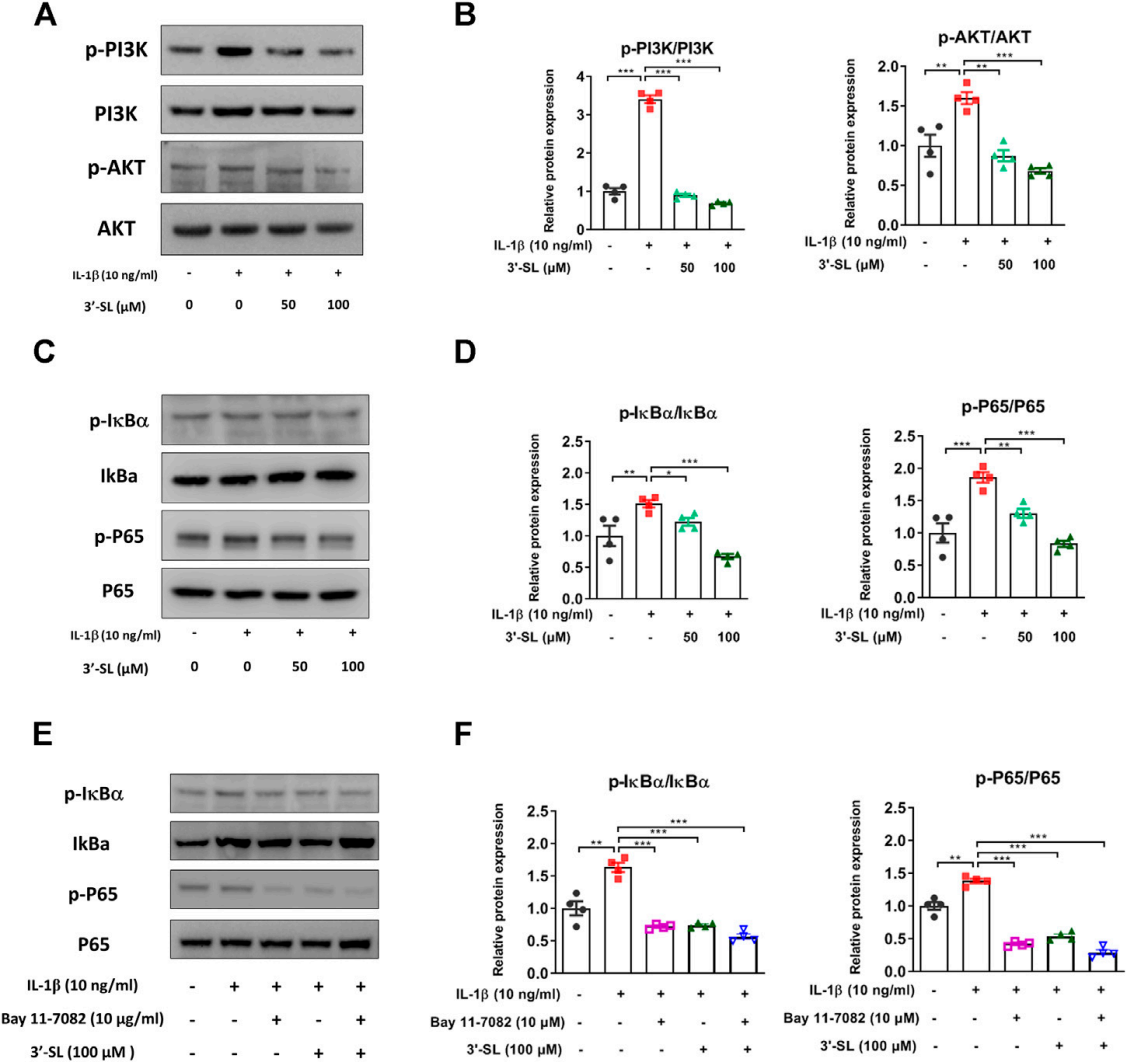
Effect of 3′-SL on IL-1β-induced PI3K/AKT/NF-κB signaling pathway in the cell model of OA. Chondrocytic cells were stimulated with or without IL-1β (10 ng/ml) for 24 h and then treated with the different concentrations of 3′-SL (50 and 100 μM) for 24 h. **(A)** The protein expression levels of p-PI3K, PI3K, p-AKT, and AKT were determined by western blot and **(B)** quantification analysis. **(C)** The protein expression levels of p-IκBα, IκBα, p-P65, and P65 were determined by western blot and **(D)** quantification analysis. **(E)** Chondrocytic cells were pretreated with Bay 11–7,082 (10 μM) for 2 h, stimulated by IL-1β (10 ng/ml) for 24 h, and then treated with the different concentrations of 3′-SL (50 and 100 μM) for 24 h. The protein expression levels of p-IκBα, IκBα, p-P65, and P65 were determined by western blot and **(F)** quantification analysis. The values are mean ± standard error of the mean. *p < 0.05, **p < 0.01, and ***p < 0.001. PI3K, Phosphatidylinositol 3-kinase; AKT, protein kinase B; NF-κB, nuclear factor kappa light chain enhancer of activated B cells.

Several studies have reported that the activation of MAPKs and phosphatidylinositol-3-kinase (PI3K)/AKT/nuclear factor kappa-light-chain-enhancer of activated B cells (NF-κB) signaling pathways plays an important role in OA progression (Hu et al., 2017; Zhang et al., 2019). Therefore, to explore whether 3′-SL can exert antioxidant protective effects, the two signaling cascades were examined. The MAPK signaling pathway was significantly activated after treatment with IL-1β in the chondrocytic cells (Figure 5, *p < 0.05, **p < 0.01, and ***p < 0.001). However, treatment with 3′-SL significantly suppressed the IL-1β-induced phosphorylation of P38, ERK, and JNK in chondrocytic cells (Figure 5, *p < 0.05, **p < 0.01, and ***p < 0.001). Moreover, 3′-SL significantly reversed the increased expression of PI3K, AKT, P65, and IκBα induced by IL-1β (Figures 6A–D, *p < 0.05, **p < 0.01, and ***p < 0.001). To further investigate the functional roles of 3′-SL, the cells were pretreated with Bay 11-7082, which significantly reduced the IL-1β-induced activation of NF-κB cascades (Figures 6E,F, **p < 0.01, and ***p < 0.001). Taken together, these findings demonstrate the suppressive effects of 3′-SL on the activated MAPK and PI3K/AKT/NF-κB signaling in IL-1β-treated chondrocytic cells, which was presumably attributed to the suppression of oxidative stress and inflammation.

## Discussion

**FIGURE 7:**
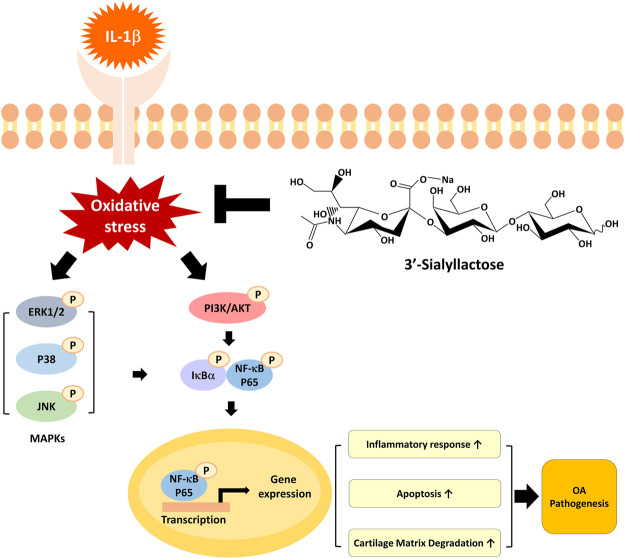
A graphical representation of the mechanism for 3′-SL in chondrocytic cells stimulated with IL-1β. 3′-SL can attenuate oxidative stress and inflammation, which induces apoptosis and cartilage degradation via the suppression of activated MAPK and PI3K/AKT/NF-κB signaling pathways in chondrocytic cells stimulated with IL-1β.

In the present study, we evaluated the underlying mechanisms of the therapeutic effects of 3′-SL using IL-1β-treated chondrocytic cells. Our data revealed that 3′-SL efficiently protects the cells from oxidative stress, inflammation, apoptosis, and cartilage matrix degradation by suppressing the activated MAPK and PI3K/AKT/NF-κB signaling pathways (Figure 7). These results provide novel insights into the therapeutic mechanisms of action of 3′-SL as a treatment for OA.

Oxidative stress is closely associated with cartilage destruction and OA progression (Sokolove and Lepus, 2013; Hu et al., 2017). The elevated production of ROS can make chondrocytes more vulnerable to oxidant-mediated cell death and lead to defective antioxidant mechanisms (Del Carlo and Loeser, 2003). Antioxidants, including SOD, catalase, and thioredoxin, are dysregulated and insufficient to detoxify ROS in OA patients (Altindag et al., 2007; Erturk et al., 2012; Gu et al., 2019; Zhong et al., 2019). Therefore, increasing the levels of these antioxidants can be used as a promising approach to prevent OA. Herein, our data revealed that 3′-SL significantly attenuated ROS production and changed intracellular redox status in the cell model of OA, as evidenced by decreased intracellular ROS production and increased oxidative stress defense system. These results demonstrated that 3′-SL can attenuate oxidative stress in IL-1β-treated chondrocytic cells.

Oxidative stress induces synovial inflammation, chondrocyte apoptosis, cartilage matrix synthesis, and intracellular signaling in OA progression (Lepetsos and Papavassiliou, 2016). Since a previous study showed that NLR family pyrin domain containing 3 (NLRP3) inflammasome activation in humans and mice is not involved in stressed-induced OA (Bougault et al., 2012), this study mainly focused on oxidative stress-related inflammation. It has been reported that nitric oxide catalyzed by iNOS and PGE2, produced from COX-2, could expedite the development of OA through ECM degradation (Sasaki et al., 1998). Moreover, IL-1β could induce an increase in iNOS and COX-2 (Daheshia and Yao, 2008; Sheu et al., 2015). Herein, the expression of iNOS, COX-2, NO, and PGE2 was usually enhanced in the cell model of OA. We found that the additional treatment with 3′-SL significantly downregulated the levels of iNOS, COX-2, NO, and PGE2. The synovial fluid and serum levels of IL-1β and IL-6 in OA patients are higher than in healthy normal subjects (Wojdasiewicz et al., 2014). In addition, it was observed that 3′-SL significantly suppressed the expression of IL-1 and IL-6 in the cell model of OA. These results demonstrated that 3′-SL can attenuate oxidative stress-derived inflammatory responses in IL-1β-treated chondrocytic cells.

Oxidative stress can cause mitochondrial apoptosis through the increased expression of the pro-apoptotic protein Bax and the decreased expression of the anti-apoptotic protein Bcl-2 (Pena-Blanco and Garcia-Saez, 2018). Our data revealed that 3′-SL significantly attenuated these changes in the expression of apoptotic proteins in IL-1β-treated chondrocytic cells. Likewise, the number of TUNEL-positive cells in the chondrocytic cells was significantly increased after IL-1β treatment. However, 3′-SL significantly decreased the number of TUNEL-positive cells. These results demonstrated that 3′-SL could reduce oxidative stress-derived apoptosis in IL-1β-treated chondrocytic cells.

Furthermore, the release of excess cartilage matrix degrading enzymes, such as MMP1, MMP3, and MMP13, is implicated in OA progression (Ahmed et al., 2005; Wang et al., 2011; Maldonado and Nam, 2013). MMPs are a family of 23 enzymes with a specific function of inhibiting the synthesis of collagen II and aggrecan, which are critical for the synthesis of matrix-related proteins in cartilage (Dahlberg et al., 2000; Yamamoto et al., 2016). Proteolysis and pathological cartilage breakdown in OA are followed by abnormal expression of MMP members (Murphy et al., 2002). Among these MMP members, MMP1, MMP3, and MMP13 are responsible for the degradation of ECM in OA articular cartilage (Yoshihara et al., 2000). In this study, IL-1β increased the expression of MMP1, MMP3, and MMP13, while the expression of matrix related proteins, such as collagen II and aggrecan, was significantly downregulated in IL-1β-treated chondrocytic cells. However, all these changes could be restored by treatment with 3′-SL. These results demonstrate that 3′-SL could attenuate oxidative stress-derived cartilage matrix degradation in IL-1β-treated chondrocytic cells.

MAPK and PI3K/AKT/NF-κB signaling pathways are key mediators of cartilage degradation and OA progression (Sun et al., 2017; Zhang et al., 2019). MAPK signaling, which consists of ERK 1/2, P38, and JNK, can transduce extracellular stimuli into the nucleus (Keshet and Seger, 2010; Sugiura et al., 2011). In addition, MAPK activation is involved in the disruption of ECM (Sondergaard et al., 2010). Subsequently, the PI3K/AKT/NF-κB signaling cascade induces increased expression of catabolic factors and can contribute to cartilage degradation (Rigoglou and Papavassiliou, 2013; Jenei-Lanzl et al., 2019). Our data showed that the phosphorylation levels of P38, ERK, JNK, PI3K, and AKT, P65, and IκBα were significantly increased in the cell model of OA. However, these activated pathways were reversed by treatment with 3′-SL. The upstream regulators of NF-κB involve the MAPK and PI3K/AKT signaling pathways (Schulze-Osthoff et al., 1997; Yum et al., 2001). Thus, we evaluated the functional kinase activities using BAY 11–7082 in IL-1β-treated chondrocytic cells. In our study, the phosphorylation of IκBα and P65 was attenuated by pre-treatment with Bay 11–7082 in IL-1β-induced chondrocytic cells. Furthermore, the inhibition of NF-kB activation by 3′-SL and Bay 11–7082 was observed in IL-1β-induced chondrocytic cells. These observations highlight the importance and need to further investigate the detailed mechanisms for the activity of other kinases. Taken together, these results reveal that the regulation of MAPK and PI3K/AKT/NF-κB signaling pathways plays a vital role in preserving the structural integrity of the matrix in the cell model of OA.

It should be note that although the chondrosarcoma cell line SW1353 is widely used as a substitute for primary adult articular chondrocytes, our results cannot be totally translated to primary OA chondrocytes considering the difference in gene expression between SW1353 and primary OA chondrocytes after treatment with IL-1β (Gebauer et al., 2005). This was the limitation of this study.

Sialyllactose is a representative human milk oligosaccharide in human breast milk. It can regulate immune homeostasis through receptor-mediated endocytosis and phagocytosis (Kim et al., 2019). Considering this and the multiple inhibitory effects of 3′-SL on IL-1β-mediated effects observed in this study, 3′-SL may mediate the receptor-mediated mechanism and could be used as a therapeutic agent for OA treatment. Further prospective studies are warranted to determine the accurate target of 3′-SL on IL-1β-induced oxidative stress and inflammation.

In conclusion, results from this study demonstrated that 3′-SL can counteract oxidative stress and inflammation via the suppression of activated MAPK and PI3K/AKT/NF-κB signaling pathways in IL-1β treated chondrocytic cells. Based on these findings, 3′-SL may be potentially used to protect against OA.

## Data Availability

The original contributions presented in the study are included in the article/Supplementary Material, further inquiries can be directed to the corresponding authors.

## Abbreviations

3′-SL, 3′-Sialyllactose; Akt, protein kinase B; DAPI, 4,6-diamidino-2-phenylindole; ECM, extracellular matrix; ERK, extracellular signal-regulated kinase; IL-1β interleukin-1β; IκBα, inhibitor of nuclear factor kappa B; JNK, c-Jun N-terminal kinase; MAPK, mitogen-activated protein kinase; NADPH, nicotinamide adenine dinucleotide phosphate; NF-κB nuclear factor kappa-light-chain-enhancer of activated B cells; OA, osteoarthritis; PI3K, phosphatidylinositol-3-kinase; qRT-PCR, quantitative Real-Time Reverse Transcription polymerase Chain Reaction; ROS, reactive oxygen species; SOD, superoxide dismutase; TUNEL, terminal dUTP nick end-labeling; XO, xanthine oxidase.
